# P037. Headache in multiple sclerosis: prevalence and clinical features in a case control-study

**DOI:** 10.1186/1129-2377-16-S1-A83

**Published:** 2015-09-28

**Authors:** Rossana Terlizzi, Elena Merli, Elena Buccellato, Giulia Giannini, Valentina Favoni, Giulia Pierangeli, Fabrizio Salvi, Pietro Cortelli, Sabina Cevoli

**Affiliations:** Department of Biomedical and NeuroMotor Sciences (DIBINEM) Alma Mater Studiorum, University of Bologna, IRCCS Institute of Neurological Sciences of Bologna, Bologna, Italy; IRCCS Institute of Neurological Sciences of Bologna, Bologna, Italy

## Background

Ten cross-sectional studies have examined a potential association between migraine and multiple sclerosis (MS); some of them found an association between the two conditions[1, 2] while five studies did not[3]. The overall incidence of migraine in MS patients ranges from 4% to 64%, but very few controlled studies has been conducted[4, 5].

## Objective

The aim of the present study was to investigate the prevalence and the clinical features of different types of headaches in subjects affected from MS respect to a control group.

## Methods

One hundred and fifty adults (F/M = 98/52; mean age 40 years) with a diagnosis of MS and 150 sex and age-matched controls (F/M = 101/49; mean age 40 years) from the general population were evaluated by means of an ad hoc semi-structured interview according to the International Classification Headache Disorders (ICHD-3-beta) criteria. All subjects filled out validated questionnaires about fatigue, Fatigue Severity Scale (FSS) and Modified Fatigue Impact Scale (MFIS). The χ2 and Kruskal-Wallis tests were used when appropriate.

## Results

The two groups differed significantly for education level and employment. Among the 150 patients with MS, 1 (0.7%) presented a radiologically isolated syndrome (RIS), 17 (11.3%) a clinically isolated syndrome (CIS), 20 (13.3%) a primary progressive form (PPMS), 96 (64%) a relapsing remitting form (RR), and 16 (10.7%) a secondary progressive form (SPMS). Headache was reported by 80 (53.3%) MS cases and 71 controls (47.3%), (p = 0.356); migraine was reported by 47 (31.33%) cases and 51 (34%) controls, tension-type headache was present in 21 (14%) MS affected vs 14 (9.33%) controls (p = 0.245). The simultaneous presence of migraine and tension-type headache was statistically higher (p = 0.002) in MS (28.8%) compared to controls (8.5%). Women with MS presented a low correlation between migraine and menstruation compared to controls while migraine normally improves during pregnancy as much as in controls (p = 0.65). The preliminary analysis of FSS and MFIS scores showed that fatigue resulted overall higher in MS patients with or without headache.

## Conclusions

Although MS patients showed a high prevalence of headache, particularly migraine, the overall prevalence was not significantly different compared to the general population. Fatigue, a well-known symptom of MS, seems to be primarily correlated to disease and poorly influenced by the presence of headache. Moreover, women with MS and migraine should be reassured regarding the possibility that their headache could improve during pregnancy as in those without MS.

Written informed consent to publication was obtained from the patient(s).
